# Development and validation of an immune‐related prognosis signature associated with hypoxia and ferroptosis in hepatocellular carcinoma

**DOI:** 10.1002/cam4.4556

**Published:** 2022-01-28

**Authors:** Chun‐Bo Yang, Han‐Xin Feng, Chao‐Liu Dai

**Affiliations:** ^1^ Department of General Surgery Shengjing Hospital of China Medical University Shenyang Liaoning China

**Keywords:** ferroptosis, hepatocellular carcinoma, hypoxia, immune

## Abstract

**Background:**

Hypoxia and ferroptosis are crucial in the occurrence and development of hepatocellular carcinoma (HCC), and they both affect the immune status of the tumor microenvironment. Previous studies have also shown a link between hypoxia and ferroptosis.

**Patients and methods:**

In all, 814 HCC cases from The Cancer Genome Atlas and Gene Expression Omnibus databases were used as the discovery cohort, and 230 HCC cases from the International Cancer Genome Consortium database were used as the validation cohort. Hypoxia subtypes and ferroptosis subtypes were identified by consensus cluster analysis according to 174 hypoxia‐related genes and 193 ferroptosis‐related genes. The prognostic signature was constructed using the Cox and LASSO regression analyses, and two risk groups were identified. A comprehensive analysis of the clinical and immune characteristics between the two risk groups was further performed.

**Results:**

Two hypoxia subtypes and two ferroptosis subtypes were distinguished and verified; subsequently, a five‐gene prognostic signature was constructed and the risk score could be acquired by the following formula: risk score = 0.0604*Expression (CA9)−0.0714*Expression (ANXA10) + 0.1501*Expression (CDC20)−0.0853*Expression (CYP7A1) + 0.0530*Expression (SPP1). Compared with the low‐risk group, the high‐risk group had a worse prognosis. The high‐risk group also showed a higher level of immune infiltration than the low‐risk group, and immune checkpoints were generally upregulated in the high‐risk group. The antigen presentation ability of the low‐risk group was poor, which may be related to the immune escape mechanism. Drug sensitivity analysis indicated that the high‐ and low‐risk groups were sensitive to tyrosine kinase inhibitors and chemotherapeutic drugs, respectively.

**Conclusion:**

The hypoxia‐, ferroptosis‐, and immune‐associated prognostic signature we constructed could stratify patients with HCC and guide precise treatment.

## Graphical abstract

A five‐gene prognostic signature associated with hypoxia and ferroptosis was identified and verified based on bioinformatics methods. Furthermore, this risk score system could stratify the immune status of patients and provide new guidance for exploring the potential mechanism of hepatocellular carcinoma development and precise treatment.

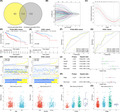

## 1 INTRODUCTION

Hepatocellular carcinoma (HCC) is the third highest cause of cancer‐related deaths worldwide, accounting for 8.3% of mortalities owing to often‐delayed diagnosis and poor prognosis.^1^ For patients with early HCC, surgery is still the main treatment, but >70% of the patients relapse within 5 years after surgical resection.^2^ Drug resistance, rapid proliferation, and the strong aggressive ability of HCC cells are reasons for the poor prognosis of HCC.^3^ Therefore, further advancements in the diagnosis and therapy are required to improve the prognosis of HCC.

Oxygen is essential for energy metabolism and biological processes. Given the rapid growth of malignant tumors and the irregular distribution of newly generated vasculature, the tumor microenvironment (TME) is in a state of hypoxia.^4^ In fact, the hypoxia in HCC is particularly serious.^5^ The drug resistance of HCC cells could increase, and the cells can evolve to a more aggressive phenotype due to hypoxia.^6^ Furthermore, the expression of E‐cadherin is reduced under hypoxic conditions, which facilitates epithelial‐to‐mesenchymal transition (EMT).^7^ Ferroptosis is a newly iron‐dependent programmed cell death pattern that is distinct from autophagy, apoptosis, and cell necrosis.^8^ The main mechanism of action of ferroptosis is via unsaturated fatty acids located on the cell membrane that undergo lipid peroxidation via catalysis of reactive ferrous iron (Fe^2+^) to produce a large amount of reactive oxygen species (ROS) that induces cell death. In addition, the decreased expression of antioxidant systems represented by glutathione peroxidase 4 (GPX4) and glutathione (GSH) is also an important reason of ferroptosis.^9^ Studies have shown that although tumor cells can escape from some forms of cell death, they are still sensitive to ferroptosis.^10^ As the first‐line drug for advanced HCC, sorafenib's main mechanism is to induce ferroptosis of HCC cells.^9^ Chang et al.^11^ confirmed that heteronemin could suppress HCC by inducing ferroptosis of HCC cells by accelerating the formation of intracellular ROS and decreasing the expression of GPX4. This evidence showed that both hypoxia and ferroptosis play an important role in HCC. Interestingly, the hypoxic environment also leads to excessive formation of ROS, but most cancer cells could still survive the oxidative stress.^4^ Using western blotting and vitality assays, Fuhrmann et al.^12^ further confirmed that hypoxia could inhibit apoptosis and ferroptosis of macrophages. However, to our knowledge, the mechanism of interaction between hypoxia and ferroptosis has not been elucidated in HCC.

In this study, we established a prognosis signature related to hypoxia and ferroptosis, and conducted a comprehensive analysis of the functional status, clinical characteristics, immune infiltration, and drug sensitivity between the high‐ and low‐risk groups. Our work explored the interaction between hypoxia and ferroptosis, which we believe could guide the treatment of HCC and improve prognosis.

## 2 PATIENTS AND METHODS

### 2.1 Data collection and processing

From the Gene Expression Omnibus (GEO) (https://www.ncbi.nlm.nih.gov/geo/) and The Cancer Genome Atlas (TCGA) (https://cancergenome.nih.gov) databases, the RNA sequencing (RNA‐seq) data of 814 HCC cases were retrieved. Four meta datasets (TCGA, GSE14520, GSE54236, GSE76427) were involved in the TCGA‐GEO cohort that was set as the discovery cohort. The FPKM values of RNA‐seq data in the TCGA cohort were further normalized as transcripts per million (TPM) values. Log‐2 transformation was performed in all expression data, and batch effects were removed using the sva R package (version: 4.0.3; https://www.r‐project.org/). Except for the GSE54236 dataset, the remaining three possessed complete clinical information. In subsequent analyses, survival time <30 days was filtered out. Based on the International Cancer Genome Consortium (ICGC) (https://dcc.icgc.org/) database, we obtained clinical information and RNA expression data of 230 Japanese patients with HCC from the ICGC‐LIRI‐JP dataset. There was no overlap of the patients between the ICGC cohort and the TCGA‐GEO cohort, and the ICGC cohort was selected for external verification.

### 2.2 Identification of the hypoxia subtypes and the hypoxia‐associated differentially expressed genes (DEGs)

We obtained 200 hallmark hypoxia genes from the Molecular Signatures Database (MSigDB version 7.4) and overlapped them with the genes in TCGA‐GEO cohort; then, 174 hypoxia‐related genes (HRGs) were selected for analysis (Table S1). Based on the HRGs expression, consensus clustering was performed in TCGA‐GEO cohorts by ConsensusClusterPlus R package with 1000 iterations and 80% resampling rate.^13^ Principal component analysis (PCA) was performed to evaluate the difference between the subtypes. To further explore the relationship between subtypes and clinical characters, Kaplan–Meier survival analysis was performed. The hypoxia‐associated DEGs between the different subtypes were identified with |log2 fold change (FC) | > 1 and a false discovery rate (FDR) < 0.05 in the limma R package.^14^

### 2.3 Identification of the ferroptosis subtypes and the ferroptosis‐associated DEGs

From the FerrDb database (http://www.zhounan.org/ferrdb/), 259 ferroptosis‐related genes (FRGs) including marker, suppressor, and driver genes were retrieved. After overlapping FRGs with the genes in the TCGA‐GEO cohorts, the expression of 193 FRGs was obtained (Table S2). According to FRG expression in the discovery cohort, consensus clustering was conducted using ConsensusClusterPlus R package, and PCA was performed to visualize the distribution of subtypes. The differences in the overall survival among subtypes were compared using Kaplan–Meier survival analysis. We selected ferroptosis‐associated DEGs between the different subtypes using the limma R package, with FDR < 0.05 and |log2 FC| > 1 set as the thresholds for differential expression.

### 2.4 Generation and validation of prognosis signature related with hypoxia and ferroptosis

A total of 114 intersection genes between hypoxia‐ and ferroptosis‐associated DEGs were identified to generate the prognosis signature. TCGA‐GEO cohort (680 patients) and ICGC cohort (230 patients) were used for identifying and validating the prognosis signature (Table S3). Univariate Cox regression analysis was performed with *p* < 0.05. To avoid overfitting, LASSO regression analysis was conducted. Subsequently, the hypoxia‐ and ferroptosis‐related signature was formulated through the multivariate Cox regression. Based on the following formula, the risk score was calculated: risk score = ∑(X_i_*Coef_i_), where *X*_i_ is the normalized expression value of the gene and Coef_i_ is the coefficient. This was applied for each patient in the four datasets and the median risk score of the TCGA‐GEO cohort was set as the threshold. Patients with higher risk scores than the threshold were considered at high risk, while those with lower risk scores than the threshold were classified as the low‐risk group.

### 2.5 Relationship of risk score with immunocyte infiltration

To verify that the risk score was associated with hypoxia status and ferroptosis, gene set enrichment analysis (GSEA) was conducted between the low‐ and high‐risk groups.^15^ Research studies have confirmed that hypoxia status and ferroptosis were closely associated with immunity; therefore, single‐sample gene set enrichment analysis (ssGSEA) was conducted based on the GSVA R package.^16^ According to gene expression, the normalized ssGSEA scores of 23 immune cells in each sample were obtained to compare the level of immune infiltration between the two risk groups (Table S4). Since immunotherapy could suppress malignancies with an excellent curative effect, we further compared the expression of 35 immune checkpoints (ICPs) between the two risk groups. The threshold for significance was *p* < 0.05.

### 2.6 Functional analyses

The DEGs between the low‐ and high‐risk groups were determined using the limma R package. FDR < 0.05 and |log2 FC| > 1 were considered to indicate significance. To further explore the biological functions and signal pathways related to the DEGs, we performed Gene Ontology (GO) and Kyoto Encyclopedia of Genes and Genomes (KEGG) pathway analyses based on the Database for Annotation, Visualization, and Integrated Discovery (DAVID 6.8; https://david.abcc.ncifcrf.gov/). FDR < 0.05 was set as the threshold.

### 2.7 Drug sensitivity analysis

We downloaded RNA‐seq and NIC‐60 drug *z*‐scores data from the CellMiner database (https://discover.nci.nih.gov/cellminer/home.do).^17^, ^18^ *Z*‐score value was positively correlated with drug sensitivity. Pearson's correlation analysis between the *z*‐score value of the FDA‐approved drug and the expression of the signature gene was performed to select sensitive therapeutic drugs in different risk groups. The thresholds were |Pearson's correlation coefficient (PCC)| > 0.3 and *p* < 0.05.

### 2.8 Screening small molecules

To screen small molecule drugs related to hypoxia and ferroptosis of HCC, we carried out Connectivity map (Cmap; https://portals.broadinstitute.org/cmap/) analysis, which can identify drugs with similar or opposite functions by comparing the DEGs under different stimulations. We loaded the up‐ and downregulated DEGs of the high‐risk group to the Cmap database, and enrichment score, ranged from +1 to −1, was obtained. A negative enrichment score means that the drug has an inhibitory effect on HCC cells, while a positive score indicates an opposite effect. The thresholds were |Enrichment score| > 0.8 and *p* < 0.05.

### 2.9 Statistical methods

Statistical analysis was conducted using the R 4.0.3 software. Categorical variables were compared using Pearson's chi‐square test or Fisher's exact test. The comparison of continuous variables were compared using Student's *t*‐test or Wilcoxon's rank‐sum test. Cox regression analysis and Kaplan–Meier analysis were performed using the survival R package. Based on the glmnet R package, LASSO regression was performed. The threshold was two‐sided *p* < 0.05.

## 3 RESULTS

### 3.1 Hypoxia‐associated subtypes and DEGs of HCC

**FIGURE 1:**
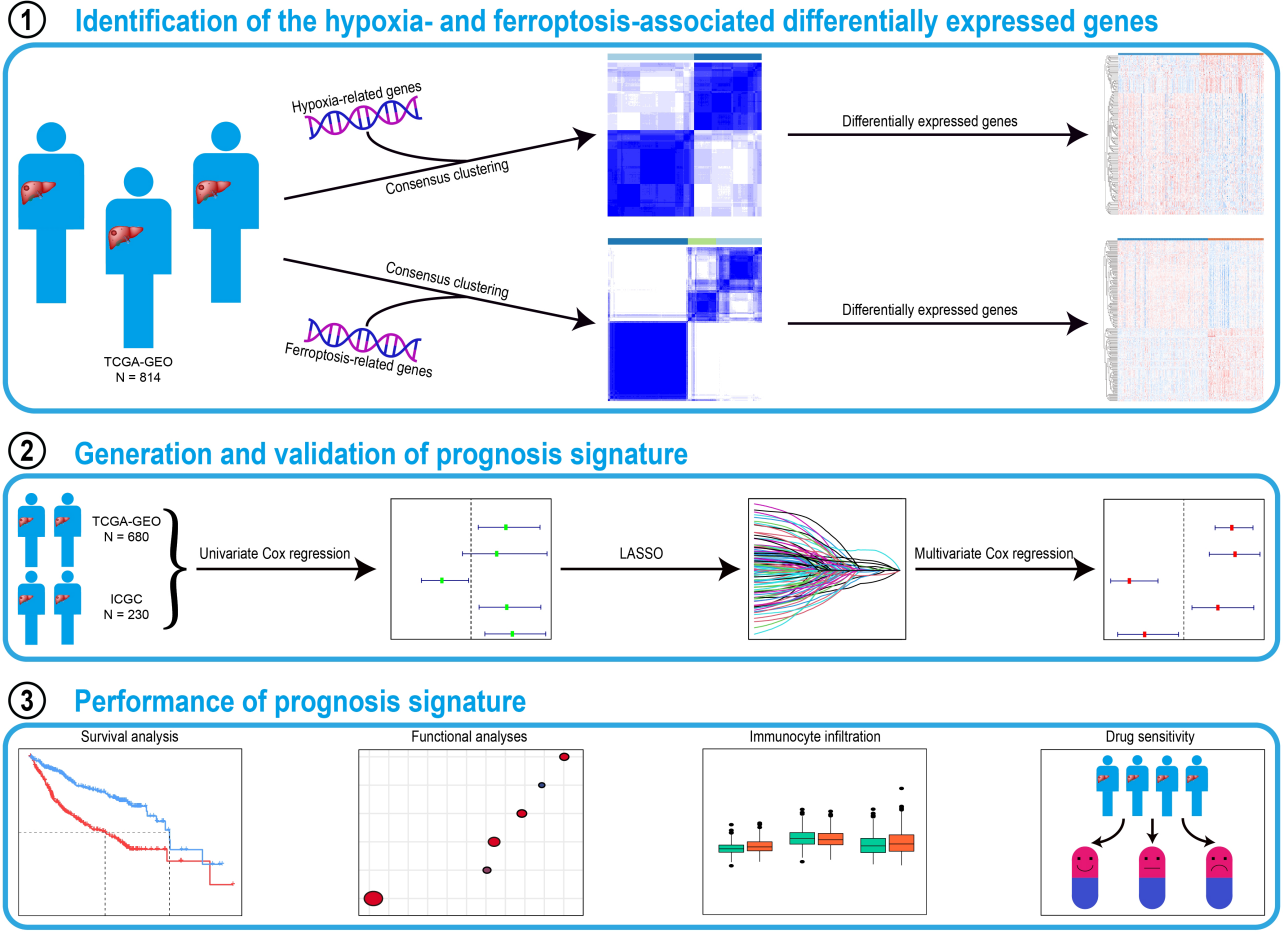
Flow chart of the study

**FIGURE 2:**
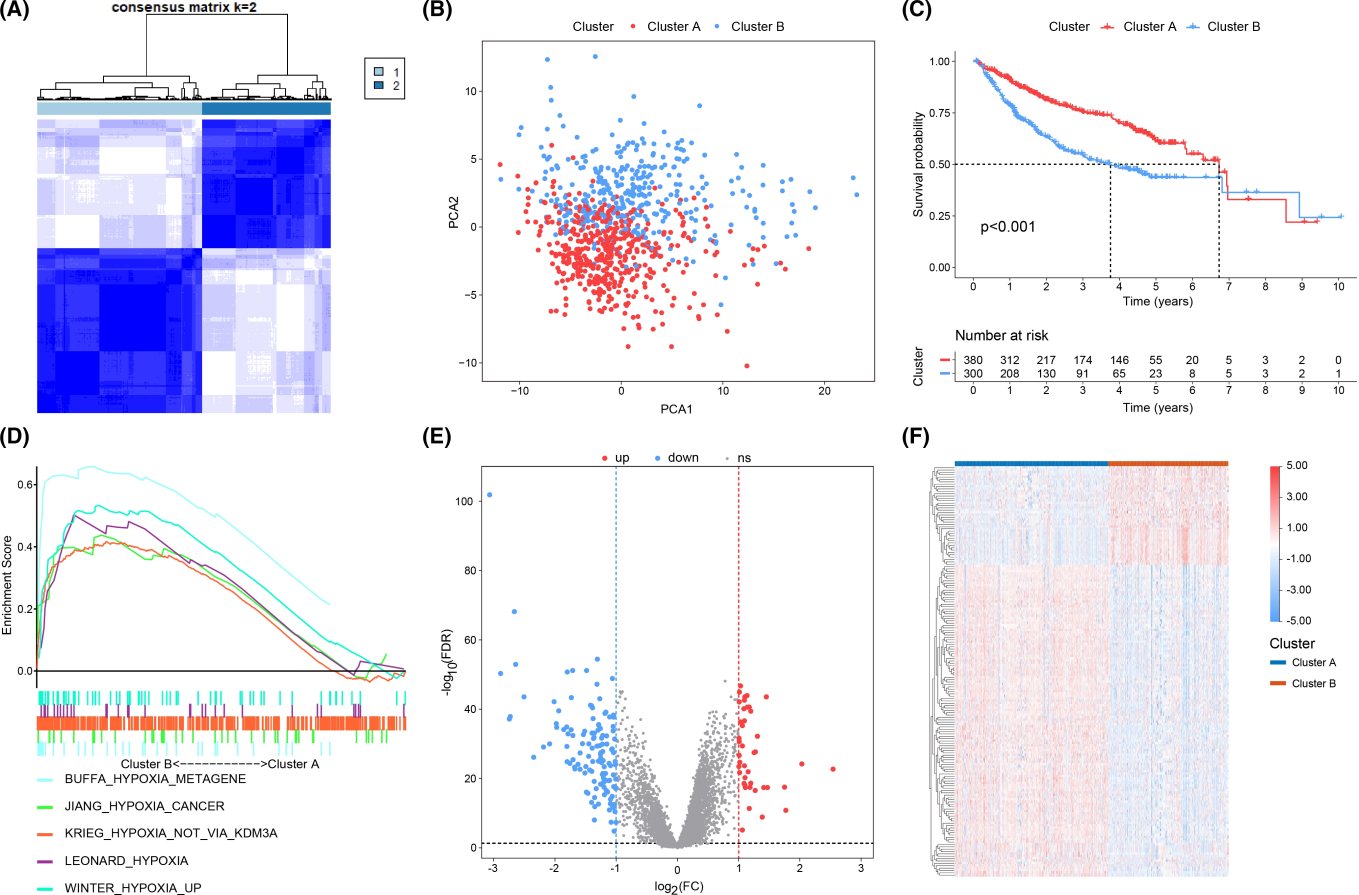
(A) The consensus matrix of all samples when *k* = 2. (B) Dot plot for two distinct clusters identified by PCA based on the expression of 174 HRGs. (C) Kaplan–Meier curves of patients in two hypoxia‐associated clusters for overall survival. (D) Five hypoxia‐associated gene sets were enriched in cluster B through GSEA. Volcano plot (E) and heatmap (F) of hypoxia‐associated DEGs between cluster B and cluster A. The blue represents low‐expressed DEGs and the red represents over‐expressed DEGs. DEGs, differentially expressed genes; GSEA, gene set enrichment analysis; HRGs, hypoxia‐related genes; PCA, principal component analysis

The flow chart was drawn in Figure 1. According to the expression of 174 HRGs, the ConsensusClusterPlus R package was used to classify 814 HCC samples of the discovery cohort into k groups (*k* = 2–9). When *k* = 2, the data acquired an optimal classification (Figure 2A). The PCA of 174 HRGs was conducted to assess the stability of proposed subtypes and revealed significant differences between the two clusters (Figure 2B). Because studies showed that hypoxia is associated with a poor prognosis, we performed survival analysis.^19^ Results showed that the survival time in cluster A is significantly longer than in B, which indicated that cluster B was in a higher state of hypoxia than cluster A (Figure 2C). To verify the high hypoxia status in cluster B, five hypoxia‐associated gene sets (BUFFA_hypoxia_metagene M34030, JIANG_hypoxia_cancer M7547, KRIEG_hypoxia_not_via_KDM3A M2469, WINTER_hypoxia_up M5466, and LEONARD_hypoxia M19622) were chosen to perform GSEA. The results revealed that five gene sets were significantly enriched in cluster B (FDR *q* < 0.25 and *p* < 0.05), which confirmed that cluster B was a high‐hypoxia subtype while cluster A was a low‐hypoxia subtype (Figure 2D). Subsequently, a total of 174 hypoxia‐associated DEGs were identified between clusters A and B, using the limma R package (Figure 2E,F; Table S5).

### 3.2 Ferroptosis‐associated subtypes and DEGs of HCC

**FIGURE 3:**
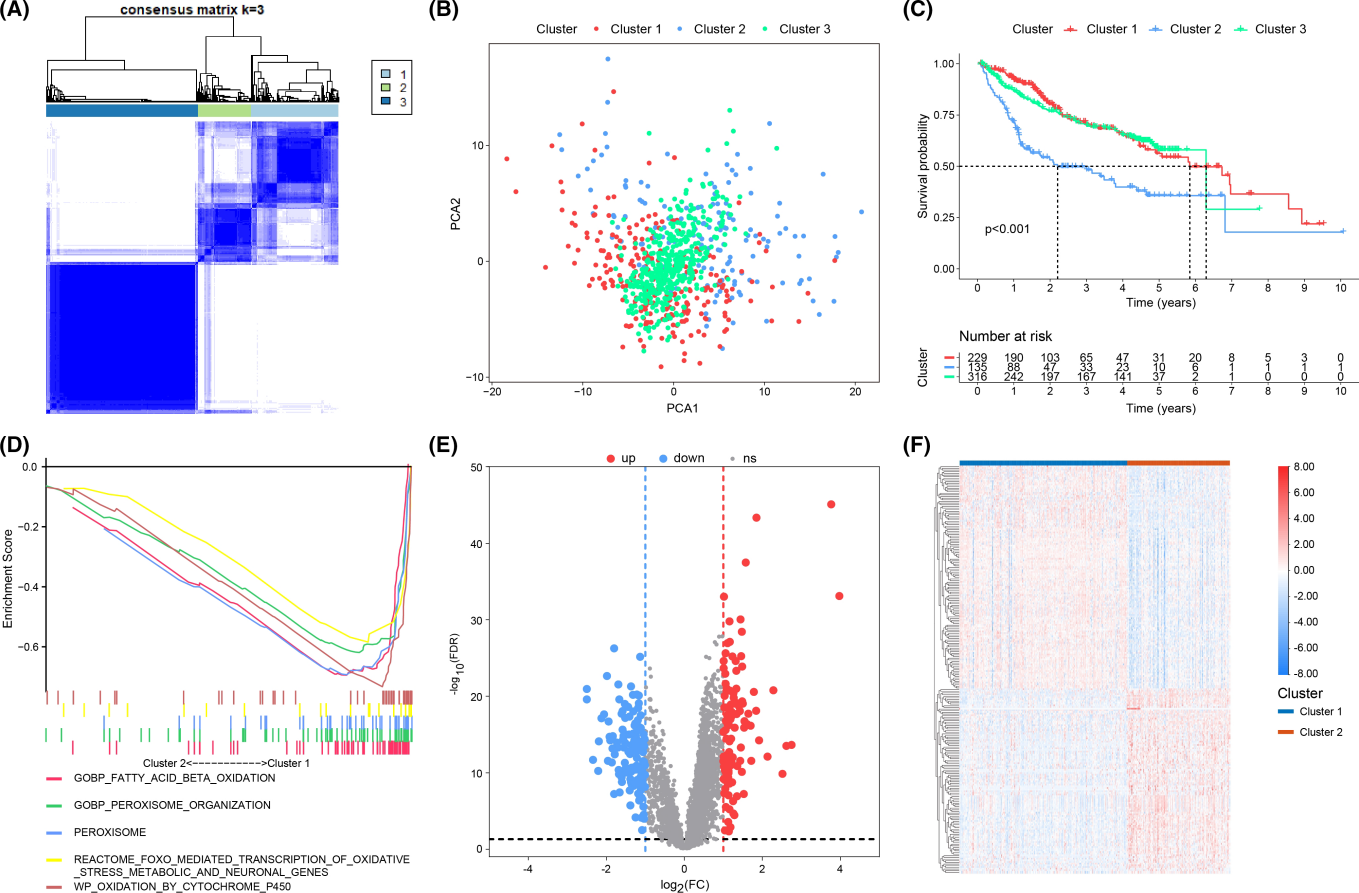
(A) The consensus matrix of all samples when *k* = 3. (B) Dot plot for three distinct clusters identified by PCA based on the expression of 193 FRGs. (C) Kaplan–Meier curves of patients in three ferroptosis‐associated clusters for overall survival. (D) Five ferroptosis‐associated gene sets were enriched in cluster 1 through GSEA. Volcano plot (E) and heatmap (F) of ferroptosis‐associated DEGs between cluster 1 and cluster 2. Blue represents low‐expressed DEGs and red represents over‐expressed DEGs. DEGs, differentially expressed genes; FRGs, ferroptosis‐related genes; GSEA, gene set enrichment analysis; PCA, principal component analysis

According to the expression of 193 FRGs, we performed consensus cluster analysis in the discovery cohort using ConsensusClusterPlus R package and 814 HCC samples were divided into k groups (*k* = 2–9). *K* = 3 could make the subtypes independent of each other that was confirmed by PCA (Figure 3A,B). Previous reports showed that ferroptosis could suppress HCC and improve prognosis.^20^ Survival analysis revealed that the prognoses of clusters 1 and 3 were significantly better than that of cluster 2, and cluster 1 possessed the best prognosis (Figure 3C). This suggested that the ferroptosis process in cluster 1 is the most active, while that in cluster 2 is the most inactive. Ferroptosis is mainly induced by the insufficient intracellular GSH and the excessive ROS produced by fatty acid oxidation.^9^ The latest research showed that peroxisome and cytochrome P450 oxidoreductase also played an important role in ferroptosis.^21^, ^22^ Based on the above findings, we selected five ferroptosis‐associated gene sets (GOBP_peroxisome_organization M3524, REACTOME_FOXO_mediated_transcription_of_oxidative_stress_metabolic_and_neuronal_genes M27941, WP_oxidation_by_cytochrome_P450 M39653, GOBP_fatty_acid_beta_oxidation M6999, and PEROXISOME M4947) to compare the ferroptosis between clusters 1 and 2, using GSEA. According to the results, the ferroptosis‐associated gene sets were significantly enriched in cluster 1 (FDR *q* < 0.25 and *p* < 0.05) (Figure 3D). The above findings demonstrated that cluster 1 was a high‐ferroptosis subtype while cluster 2 was a low‐ferroptosis subtype. Therefore, we analyzed the differences of genes between cluster 1 and cluster 2, and a total of 247 ferroptosis‐associated DEGs were identified (Figure 3E,F; Table S6).

### 3.3 Construction and verification of hypoxia‐ and ferroptosis‐associated prognosis signature

**FIGURE 4:**
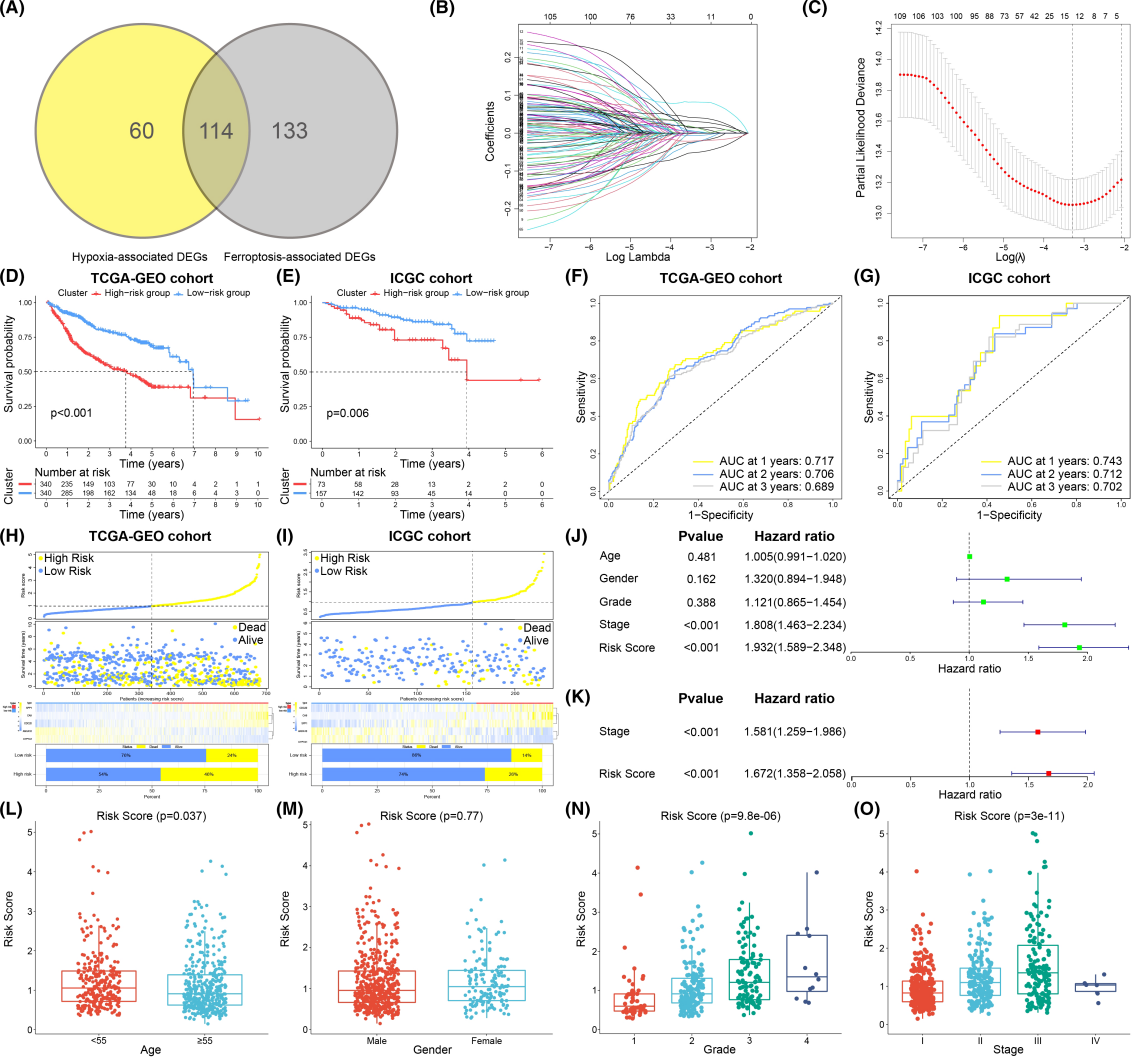
(A) Venn diagram displaying the intersection genes of the hypoxia‐associated DEGs and the ferroptosis‐associated DEGs. (B) LASSO regression coefficient profile of the 112 intersection genes. (C) LASSO deviance profile of the 112 intersection genes. Survival curves of the two subtypes in the TCGA‐GEO cohort (D) and ICGC cohort (E). ROC curves for the 1‐, 2‐, and 3‐year survival time based on the risk score in the TCGA‐GEO cohort (F) and ICGC cohort (G). Distribution of risk score, overall survival, gene expression, and survival status between low and high‐risk groups in the TCGA‐GEO cohort (H) and ICGC cohort (I). Univariate (J) and multivariate (K) Cox regression analysis of risk score and clinical factors. (L–O) Correlation of the prognostic signature with clinical characteristics, such as age (L), sex (M), grade (N), and clinical stage (O). DEGs, differentially expressed genes; ROC, receiver operating characteristic

After overlapping the hypoxia‐ and ferroptosis‐associated DEGs, 114 intersection genes were screened for the construction of prognosis signature (Figure 4A). Univariate Cox regression analysis screened out 112 prognostic genes (Table S7). To avoid overfitting of the signature, LASSO regression analysis was conducted, and 13 genes were identified for multivariate Cox regression analysis (Figure 4B,C). Ultimately, five genes were incorporated into the best prognosis signature, and the following formula could be used to acquire the risk score: Risk score = 0.0604 × Expression (CA9)−0.0714 × Expression (ANXA10) + 0.1501 × Expression (CDC20)−0.0853 × Expression (CYP7A1) + 0.0530 × Expression (SPP1). Low‐ or high‐risk group was determined based on the median risk score. Survival analysis confirmed that the low‐risk group had a better prognosis than the high‐risk group (*p* < 0.05) (Figure 4D,E). According to the survival information, receiver operating characteristic (ROC) curves were drawn in the TCGA‐GEO and ICGC cohorts (Figure 4F,G). We assessed the accuracy of the risk score through the values of area under the curve (AUC). The AUCs of the ROC curves related to survival rates in the TCGA‐GEO cohort (AUC at 1, 2, 3 years: 0.717, 0.706, 0.689, respectively) and in the ICGC cohort (AUC at 1, 2, 3 years: 0.743, 0.712, 0.701, respectively) were calculated. The heatmaps of genes and the distributions of survival status and risk score were visualized (Figure 4H,I). With the rising of the risk score, the survival time of patients was shortened, and the expression of CA9, CDC20, and SPP1 were increased. However, ANXA10 and CYP7A1 were downregulated in the high‐risk group. Clinical features (age, sex, grade, and stage) and risk scores were together analyzed using univariate and multivariate analysis. The results verified that the hypoxia‐ and ferroptosis‐associated risk score could act as an independent prognosis factor (Figure 3J,K). Based on the clinical information, we confirmed that the risk score was correlated with the age, grade, and TNM stage statistically (Figure 4L–O).

### 3.4 Relationship of risk score with immunocyte infiltration

**FIGURE 5:**
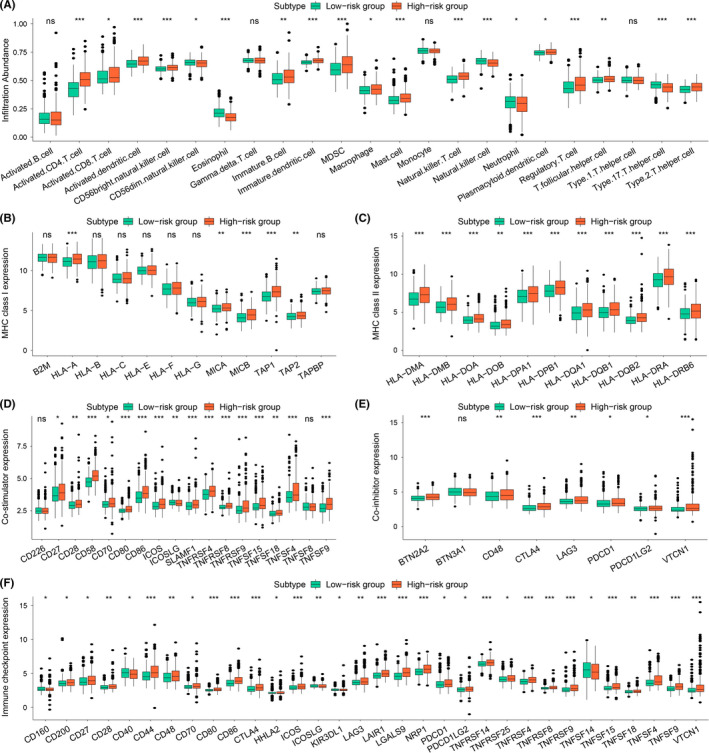
(A) The infiltration differences of immune cells between different risk groups. The difference of mRNA expression for MHC molecules (B, C), co‐stimulators (D), co‐inhibitors (E), and immune checkpoints (F) between different risk groups. The statistical *p* values were displayed as: ns, *p* > 0.05; *p < 0.05; **p < 0.01; ***p < 0.001. MHC, major histocompatibility complex; ns, no statistical difference

Based on ssGSEA, difference analysis was conducted to further compare the differences of immunocyte infiltration between the two risk groups (Figure 5A). The content of most immune cells was significantly different between the two risk groups, and most immune cells of the high‐risk group were upregulated. Based on the expression of ICPs and the antigen presentation capacity, we further analyzed the immune escape mechanisms of different groups. The expression of major histocompatibility complex (MHC) Class I and MHC Class II molecules was significantly downregulated. The low‐risk group possessed a poor antigen presentation capacity (Figure 5B,C).^23^ Meanwhile, we found that ICPs, coinhibitory, and costimulatory molecules in the high‐risk group were significantly upregulated, which might be associated with immune escape (Figure 5D–F). These indicated that HCC with high‐risk scores is more suitable for treatment with immune checkpoint inhibitors, which provides a direction for individualized immunotherapy of HCC.

### 3.5 Evaluating the connection of risk score with hypoxia and ferroptosis

**FIGURE 6:**
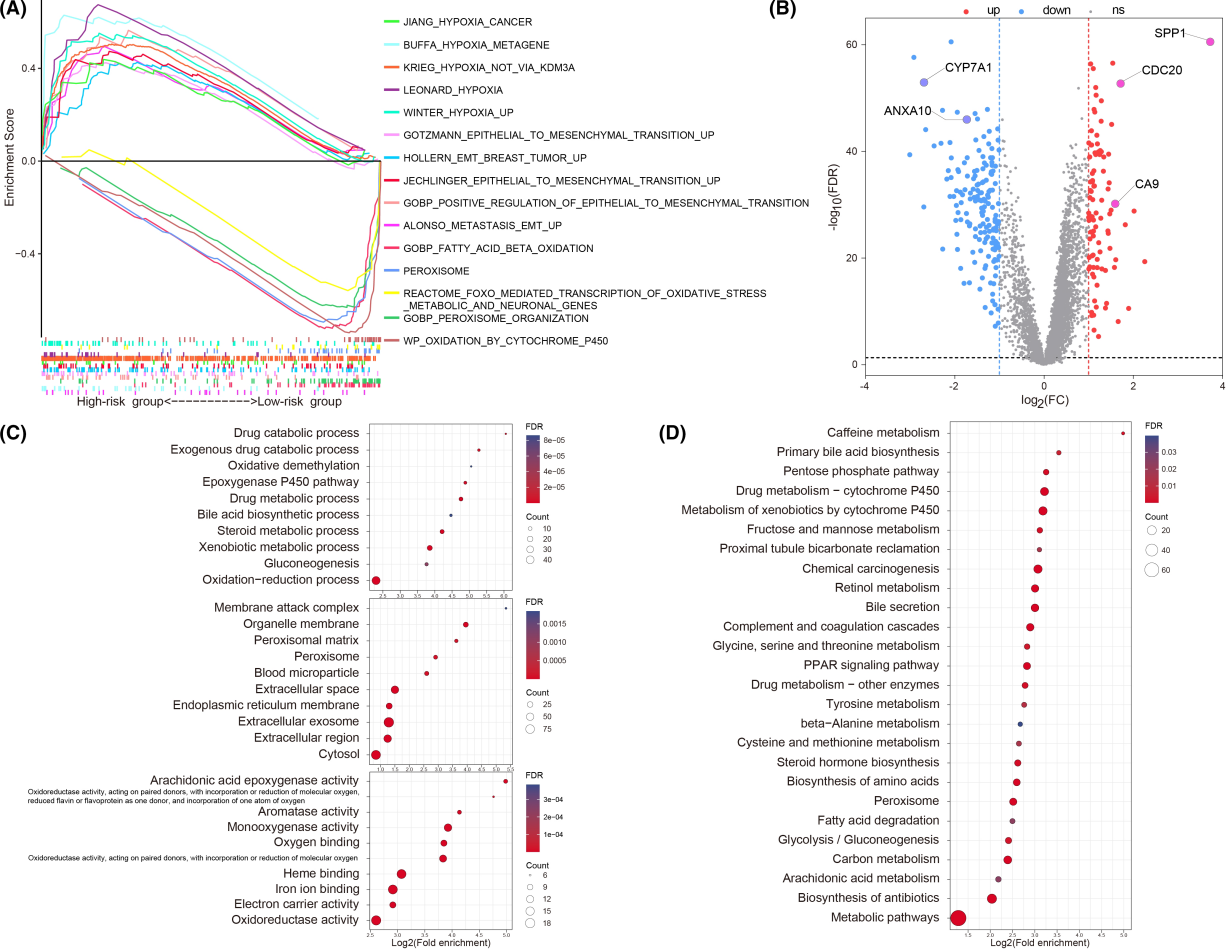
(A) Five hypoxia‐associated gene sets and five EMT‐associated gene sets were enriched in the high‐risk group, while five ferroptosis‐associated gene sets were enriched in the low‐risk group through GSEA. (B) Volcano plot of the DEGs between different risk groups. Blue represents low‐expressed DEGs and red represents over‐expressed DEGs. Representative results of the Gene Ontology (C) and Kyoto Encyclopedia of Genes and Genomes (D) analyses between different risk groups. EMT, epithelial‐to‐mesenchymal transition; DEGs, differentially expressed genes; GSEA, gene set enrichment analysis

To validate the relation of risk score with hypoxia and ferroptosis, GSEA was performed using the previous five hypoxia‐associated and five ferroptosis‐associated gene sets, respectively. The results revealed that hypoxia‐associated gene sets were significantly enriched in the high‐risk group, while ferroptosis‐associated gene sets were significantly enriched in the low‐risk group (FDR *q* < 0.25 and *p* < 0.05) (Figure 6A). Furthermore, studies showed that hypoxia could induce epithelial cells to undergo EMT.^24^ Thus, five EMT‐related gene sets (JECHLINGER_epithelial_to_mesenchymal_transition_UP M1406, HOLLERN_EMT_breast_tumor_up M617, ALONSO_metastasis_EMT_up M8191, GOBP_positive_regulation_of_epithelial_to_mesenchymal_transition M10621, and GOTZMANN_epithelial_to_mesenchymal_transition_up M1373) were selected for GSEA. We confirmed that in the high‐risk group, EMT‐associated gene sets were also significantly enriched (FDR *q* < 0.25 and *p* < 0.05) (Figure 6A). These findings verified that the high‐risk group is at a higher state of hypoxia with lower activity of ferroptosis and low‐risk group is at a lower state of hypoxia with higher activity of ferroptosis, which indicated that our risk score could accurately assess hypoxia state and ferroptosis of HCC.

### 3.6 Functional analyses

Based on the limma R package, 164 downregulated DEGs and 94 upregulated DEGs were screened out in the high‐risk group (Figure 6B). To further explore the related functions of DEGs, GO and KEGG analyses were conducted. The GO enrichment analysis results showed enrichment in 20 molecular function terms, 14 cellular component terms, and 31 biological process terms (Table S8). The top 10 enriched terms for each classification are displayed in Figure 6C. It can be confirmed that the DEGs were significantly enriched in biological processes such as redox, iron ion binding, and oxygen binding. A total of 26 enriched pathways were obtained through KEGG analysis, and small molecule metabolism and PPAR signaling pathway showed enrichment of the DEGs (Figure 6D; Table S9).

### 3.7 Evaluating the relationship between drug activity and signature genes

**FIGURE 7:**
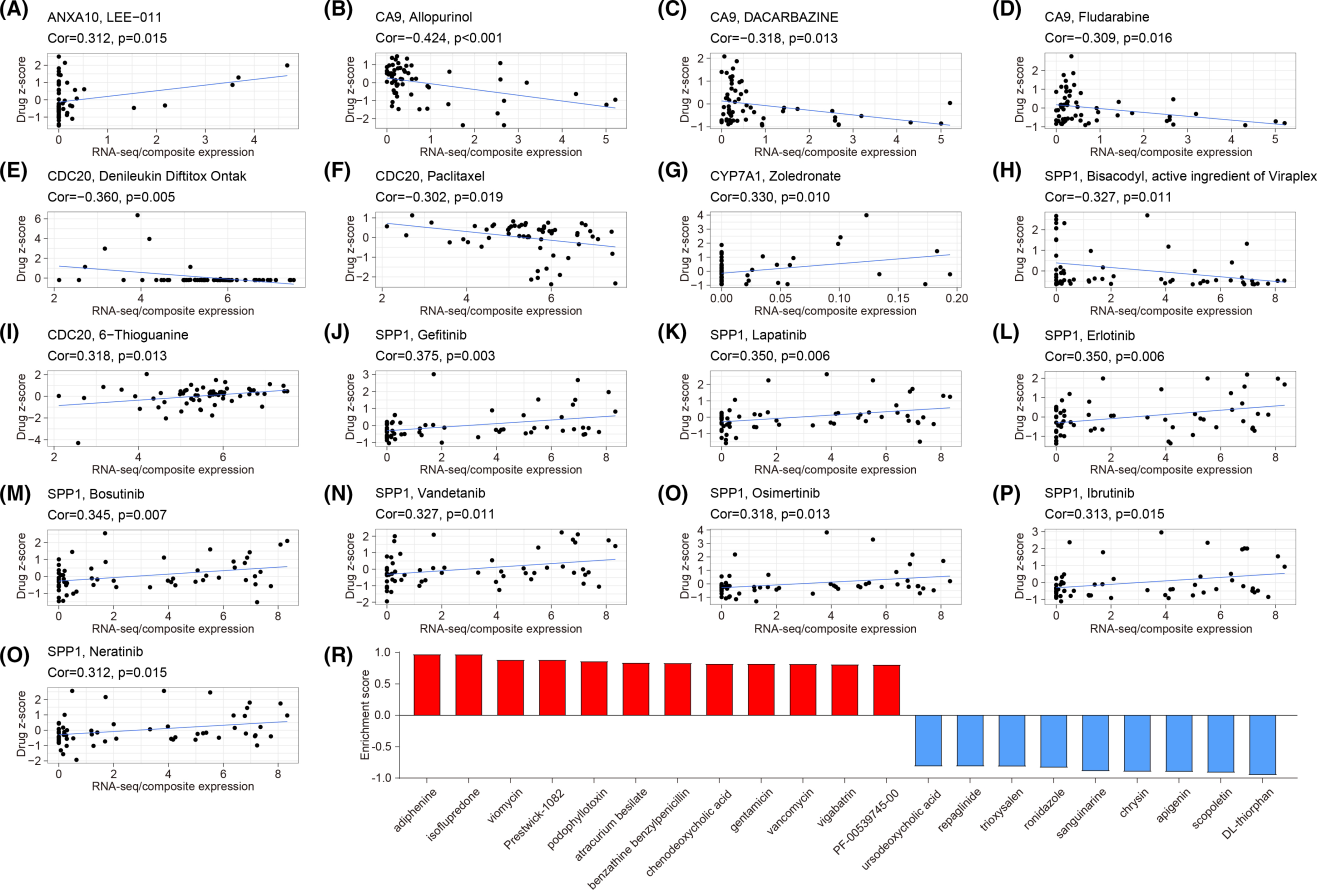
(A–Q) Drug sensitivity analyses based on the CellMiner database through the correlation analysis of drug *z*‐score with gene expression. The independent sample is shown as a black dot. The linear regression is shown as the blue line. (A–H) The drugs that the low‐risk group was sensitive to. (I–Q) The drugs that the high‐risk group was sensitive to. (R) Small molecules associated with the risk score. The red represents molecules that strongly suppress HCC in the low‐risk groups. The blue represents molecules that strongly suppress HCC in the high‐risk groups. HCC, hepatocellular carcinoma

To screen potential drugs for the treatment of HCC, the relationship between drugs' z‐score values and the expression of signature genes were analyzed. With the threshold as |PCC| > 0.3 and *p* < 0.05, a total of 17 drugs were identified to be significantly correlated with signature genes (Figure 7A–Q). When the gene was upregulated in the population and the gene expression was positively correlated with the *z*‐score value, the population was considered sensitive to the drug. However, when the gene was downregulated and the gene expression was negatively correlated with the *z*‐score value, the population was considered insensitive to the drug. Thus, we summarized that the cases in the low‐risk group were more sensitive to LEE‐011, allopurinol, dacarbazine, fludarabine, denileukin diftitox (Ontak), paclitaxel, zoledronate, and bisacodyl, while the samples in the high‐risk group were more sensitive to 6‐thioguanine, gefitinib, lapatinib, erlotinib, bosutinib, vandetanib, osimertinib, ibrutinib, and neratinib.

### 3.8 Candidate small molecules associated with the risk score

We loaded 94 upregulated and 164 downregulated DEGs to the Cmap database, and a total of 21 small molecule drugs associated with the risk score were screened out (Figure 7R). Among these 21 small molecule drugs, nine (such as dl‐thiorphan, scopoletin, and apigenin) had negative enrichment scores, which indicated that they have a strong inhibitory effect on HCC cells in high‐risk groups. The enrichment scores of 12 small molecules (such as adiphenine, isoflupredone, viomycin) were positive, indicating that they have a strong inhibitory effect on HCC cells in low‐risk groups.

## 4 DISCUSSION

According to the latest statistics, there are approximately 905,677 new cases of HCC and 830,180 deaths each year.^1^ Heterogeneity is the main reason why malignancies cannot be treated effectively, and the heterogeneity of HCC is particularly high; thus, finding a convenient method that can guide individualized and precise treatment for patients with HCC is clinically paramount.^25^, ^26^ As a hallmark of TME in HCC, although hypoxia is a harsh environment, the proliferation and metastasis of HCC cells could be accelerated through a series of compensation processes mediated by HIF signaling pathways, which causes worse prognosis.^27^ Ferroptosis has been proven to be significantly related to tumor suppressor effect and immune status, and inducing ferroptosis of HCC cells is considered an effective way to treat HCC.^28^, ^29^ Recent studies have shown that the ferroptosis of tumor cells under hypoxia was inhibited, but the specific molecular regulatory mechanism is still unclear.^12^

In our research, high‐ and low‐hypoxia subtypes and high‐ and low‐ferroptosis subtypes were identified. Survival analysis revealed that the high‐hypoxia subtype possessed a worse prognosis, while the prognosis of the high‐ferroptosis subtype was better. In the enrichment analysis, the hypoxia‐ and ferroptosis‐related gene sets were utilized to validate the function of the subtypes. The results showed that the hypoxia‐related gene sets were significantly enriched in the high‐hypoxia subtype, and the ferroptosis‐related gene sets were significantly enriched in the high‐ferroptosis subtype. Based on the screened hypoxia‐ and ferroptosis‐associated DEGs, a five‐gene prognostic signature was constructed. Two risk groups were identified based on the risk score. Survival analysis confirmed that the low‐risk group had a significantly better prognosis. Through functional verification, it was confirmed that the low‐risk group was at a low hypoxia state accompanied by high ferroptosis, while the high‐risk group was at a high hypoxia state with low ferroptosis. Univariate and multivariate Cox regression analysis showed that the risk score was confirmed as an independent predictor. Meanwhile, the degree of immune infiltration and drug sensitivity of low‐ and high‐risk groups also showed obvious heterogeneity. These results indicate that our prognostic signature could effectively assess the hypoxia state and ferroptosis in HCC, which guided the individualized precision treatment for patients with HCC and further exploration of the mechanisms between hypoxia and ferroptosis.

There were significant differences in risk scores among different clinical characteristics. The risk score increased accompanied by an increase in the grade and stage of HCC, which also meant an increase in the degree of hypoxia and suppression of ferroptosis. Although numerous studies believed that the proportion of advanced HCC in young patients was higher than that in elderly patients, there was still a controversy on the impact of age in the survival of HCC.^30^, ^31^, ^32^ A recent study revealed that the 5‐year survival rate of young patients with HCC was 15% lower than that of elderly patients (*p* = 0.007).^33^ In our study, the younger group showed a higher risk score which might explain the poor prognosis of young patients with HCC and suggested that age might be a negative predictor for survival of HCC. Since hypoxia and ferroptosis are closely associated with the immune response of HCC cells, the level of immune cell infiltration between the two risk groups was compared.^34^, ^35^ Although our study found that immune cells in the high‐risk group were notably higher, the infiltration level of natural killer cells (NKCs) was significantly increased in the low‐risk group. According to reports, NKCs can induce ferroptosis via production of H_2_O_2_ catalyzed by NADPH oxidase 2 (NOX2), and under hypoxic conditions, the cytotoxicity of NKCs could be inhibited.^28^, ^36^ These might partially explain the better prognosis of the low‐risk group. The relationship between the immune escape mechanism and risk score was further explored, and the results showed that the immune escape of high‐risk groups might be related to the overexpression of ICPs, while the immune escape of low‐risk groups might be related to a poor antigen presentation ability. These findings indicated that hypoxia and ferroptosis could regulate TME in HCC, and our signature could assess the immune status and guide the immunotherapy of patients with HCC.

Hypoxia‐induced drug resistance is one of the reasons for the poor effect of chemotherapy on HCC.^6^, ^37^ Paclitaxel, dacarbazine, and fludarabine are usually unsatisfactory in the chemotherapy of hypoxic tumors.^38^, ^39^, ^40^ Our results showed that the low‐risk group is sensitive to chemotherapeutic drugs, which might be associated with the low hypoxic state in the low‐risk group. Currently, tyrosine kinase inhibitors (TKIs) have become an indispensable part of the treatment for advanced HCC.^41^ In addition to sorafenib and lenvatinib, there are still many new TKIs appearing.^42^ Through transcriptome analysis and immunoblotting, Nagpal et al. confirmed that neratinib could suppress cancer by promoting ferroptosis of cancer cells.^43^ Siramesine is a lysosomotropic agent and can be combined with lapatinib to induce ferroptosis via upregulating transferrin.^44^ In this study, the high‐risk group showed high sensitivity to TKIs (gefitinib, lapatinib, erlotinib, bosutinib, vandetanib, osimertinib, ibrutinib, and neratinib). The combination of TKIs and lysosomotropic agents might be an effective treatment strategy for HCC with a high‐risk score. Meanwhile, we obtained 21 small molecule compounds that could specifically suppress HCC with different risk scores based on the Cmap analysis. These results provided a theoretical basis for individualized precision treatment of HCC.

Our research has some limitations. First, because of the heterogeneity within the tumor, the TME may be different in different positions of the same tumor. Second, the trials of drug sensitivity were still based on theoretical data analysis and further prospective studies are needed to certify the results.

## 5 CONCLUSIONS

In summary, a five‐gene prognostic signature associated with hypoxia and ferroptosis were identified and verified. In addition, this risk score system could stratify the immune status of patients. These results provided new guidance for exploring the potential mechanism of HCC development and precise treatment.

## CONFLICT OF INTEREST

The authors declare that they have no competing interest.

## ETHICAL APPROVAL STATEMENT

All data of this study were public and required no ethical approval.

## Supporting information


Table S1

Table S2

Table S3

Table S4

Table S5

Table S6

Table S7

Table S8

Table S9

## Data Availability

All data analyzed or generated during this study are available upon reasonable request.
